# Enhanced Intestinal Permeability of Bufalin by a Novel Bufalin-Peptide-Dendrimer Inclusion through Caco-2 Cell Monolayer

**DOI:** 10.3390/molecules22122088

**Published:** 2017-11-29

**Authors:** Chi-on Chan, Jing Jing, Wei Xiao, Zhexu Tan, Qiuyue Lv, Jingyu Yang, Sibao Chen

**Affiliations:** 1 State Key Laboratory of Chinese Medicine and Molecular Pharmacology (Incubation), Hong Kong Polytechnic University Shenzhen Research Institute, Shenzhen 518057, China; on.chan@polyu.edu.hk; 2 School of Pharmacy, University of Queensland, Woolloongabba QLD 4072, Australia; jing.jing1@uqconnect.edu.au; 3 Institute of Medicinal Plant Development, Chinese Academy of Medical Sciences and Peking Union Medical College, Beijing 100193, China; wxiao@implad.ac.cn (W.X.); tftzx1012@126.com (Z.T.); 18813122696@163.com (Q.L.); 4 Shenzhen Pharmacists Association, Shenzhen 518024, China; szpa2012@126.com

**Keywords:** bufalin, peptide-dendrimer, bufalin-peptide-dendrimer inclusion, Caco-2 cell monolayer, intestinal permeability, apparent permeability coefficient

## Abstract

Bufalin (BFL) has excellent physiological activities such as defending tumors, improving cardiac function, and so on. However, due to its poor water-solubility and bioavailability, the clinical application of BFL remains limited. In order to improve bioavailability of BFL, in our previous research, a novel peptide-dendrimer (PD) was synthesized and applied to encapsulate BFL. In the present study, we investigate the absorption property and mechanism of BFL in free form and BFL-peptide-dendrimer inclusion (BPDI) delivery system by using the Caco-2 cell monolayer model in vitro. The apparent permeability coefficient (*P*_app_) values of BFL in free or BPDI form were over 1.0 × 10^−6^ cm/s. Meanwhile, their almost equal bi-directional transport and linear transport percentage with time and concentration course indicated that BFL in both forms was absorbed mainly through passive diffusion. The most important result is that the *P*_app_ values of BFL increased about three-fold more BPDI than those of its free form, which indicated the intestinal permeability of BFL could be improved while BFL was encapsulated in BPDI form. Therefore, PD encapsulation may be a potential delivery system to increase the bioavailability of BFL.

## 1. Introduction

**Figure 1:**
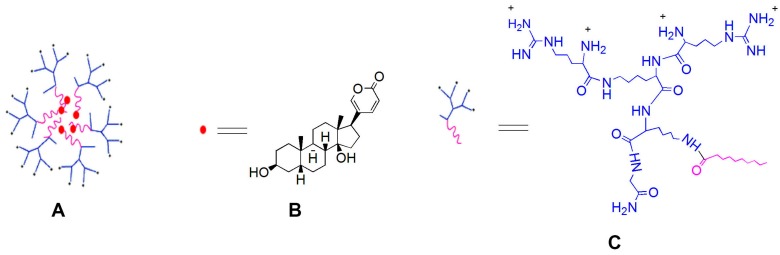
Molecular structure of bufalin-peptide-dendrimer inclusion (**A**); bufalin (**B**) and peptide-dendrimer (**C**).

Toad venom is the dried secretion from the posterior auricular glands or skin glands of *Bufobufogargarizans* Cantor or *Bufomelanostictus* Schneider [1]. Due to its unique properties—such as detoxification, pain relief, and inducing resuscitation [2]—toad venom has been used as traditional Chinese medicine since around 581 BC (Tang Dynasty)for the treatment of furuncle and carbuncle, sore throat, mental confusion caused by heatstroke and abdominal pain, vomiting, and diarrhea [3]. Bufalin (BFL) (Figure 1B), the major bufanolide steroid in toad venom, has demonstrated a variety of biological activities including tumor inhibition, enhancing cardiac function, exciting respiration, anti-inflammation, boosting immunity, and providing local anesthesia [4]. However, due to the highly hydrophobic structure, BFL is a poorly water-soluble drug and with low bioavailability, which limits its application in clinic [5]. Hence, improving intestinal absorption and bioavailability of BFL by developing an appropriate drug delivery system or drug carrier is crucial for enlarging its range of therapeutic applications.

Solid dispersions, polymer nanoparticles, lipid nanoparticles, micelles, and microemulsions have been developed and applied for delivery of poorly water-soluble drugs [5]. Among them, a dendrimer-based delivery system has shown good application prospects as a drug target vector and controlled-release carrier [6,7,8]. In recent years, asymmetric peptide dendrimer (PD) has attracted more attention and has been more properly applied for drug delivery due to its more advantages in terms of excellent biocompatibility, low cytotoxicity, and protease-hydrolysis resistance while compared to the low generation commercial PAMAM dendrimers [9,10,11]. In brief, asymmetric PD is highly branched with three-dimensional space and provides many hydrophobic cavities [12]. The poorly water-soluble small molecules can be attached to the dendrimers according to physical inclusion or noncovalent combination, and hence the water solubility as well as intestinal absorption can be increased [13,14]. With respect to the enhancing effects of PD on the bioavailability of small molecules, there have been a handful of reports. For instance, a synthesized PD with arginine as the terminal amino acid has been reported to enhance the deposition and permeation of 5-fluorouracil across human epidermis [15]. Meanwhile, PDs were also efficient on improving the antigen-binding capacity of antibodies by site-specifically conjugating to antibodies, and therefore were applied for antibodies delivery [16].

To improve the bioavailability of BFL, in our previous study, an asymmetric amino acid-based PD (Figure 1C) was synthesized and applied to encapsulate BFL, and eventually constructed the bufalin-peptide-dendrimer inclusion (BPDI) (Figure 1A). In the present paper, we investigate and compare the permeation properties of BFL when it was in free form or in BPDI using the well-accepted human colon adenocarcinoma (Caco-2) cell monolayer model [17], finally to predict if BPDI is useful to improve the absorption and availability of BFL.

## 2. Results

### 2.1. Cytotoxicity of BPDI on Caco-2 Cells

The viability of Caco-2 cells were measured after treated for 180 min with Hanks’ balanced salts solution (HBSS) (negative control), paclitaxel (positive control, 25, 50, 75 and 100 μM), BFL (50, 75, 100 and 125 μM) and BPDI (containing BFL 50, 75, 100 and 125 μM), respectively. Results (Table 1) showed that the viabilities after treatment by BFL or BPDI were over 95%, which indicated that they were no-toxic to Caco-2 cells at these concentrations for 180 min. Hence, those concentrations of BFL and BPDI were chosen as the test concentrations.

### 2.2. Validation of Caco-2 Monolayer Model

After seeding, the transepithelial electrical resistance (TEER) values of the monolayer developed in this study increased steadily over time, and were above 500 Ω/cm^2^ on day 21. The apparent permeability coefficient (*P*_app_) values of propranolol and atenolol tested with the monolayer were (5.17 ± 0.16) × 10^−5^ cm/s and (6.86 ± 1.15) × 10^−7^ cm/s, respectively, which were closely consistent with the acceptable values reported in the literature [18,19,20]. Thus, the Caco-2 cell monolayer model established was validated for the assessment of the intestinal absorption potential of BFL and BPDI.

### 2.3. Transmembrane Transport of BFL and BPDI

**Figure 2:**
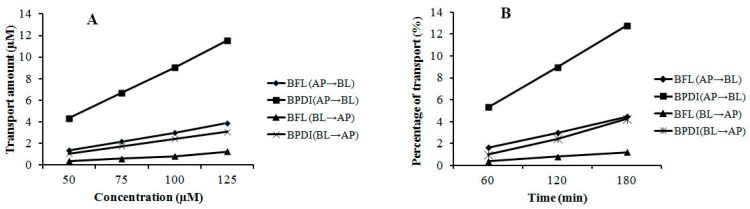
Transports of BFL and BPDI across Caco-2 cell monolayer. (**A**) The transport amounts at 120 min with different concentrations (*n* = 3); (**B**) The percentages of transports in different time points with a concentration of 75 μM (*n* = 3).

The transmembrane transports of BFL and BPDI were evaluated by using the validated Caco-2 cell monolayer model. As shown in Figure 2, either in free BFL or in BPDI, the bidirectional transport percentages of BFL increased approximately linearly with time (Figure 2A), and their transport amounts increased with concentration in the range of 50–125 μM in an approximately linear manner at 120 min (Figure 2B). Meanwhile, it can be observed that the transports of BFL in the BPDI form increased while compared to those in free BFL.

The *P*_app_ values of bi-directional transport of BFL and BPDI were summarized in Table 2 and Table 3. In general, BFL and its formulation BPDI showed moderate transport due to a *P*_app_ value of 1–10 × 10^−6^ cm/s. *P*_app_ value of BPDI ranged from 4.18–5.24 × 10^−6^ cm/s, which was about three-fold increment of that of freeBFL (1.46–1.62 × 10^−6^ cm/s). Furthermore, *P*_app_ values of free BFL or in BPDI have no significant variation with the change of concentration or transport time.

By the way, the *P*_app_ of BFL and BPDI in the apical-to-basolateral direction (*P*_app AP→BL_), as well as that in the basolateral-to-apical direction (*P*_app BL→AP_) were compared respectively to explore the possible transporting mechanism. As shown in Table 2, the ratios of *P*_app AP→BL_ to *P*_app BL→AP_, were larger than 1 both in free BFL and BPDI. Nevertheless, these ratios of BFL and BPDI were very similar each other.

### 2.4. The Validation of HPLC Analytical Methods

The HPLC-DAD method was validated in terms of linearity, precision, accuracy. Results showed that the linear regression equation was obtained as *y* = 12.295*x* + 23.295 with *r*^2^ of 0.9966. The linear range was from 0.2 µmol/L to 20 µmol/L. The limit of detection (LOD) and limit of quantitation (LOQ) were 0.05 µmol/L and 0.17 µmol/L, respectively. The relative standard deviations (RSDs) of intra-day and inter-day precisions were of 1.25% and 2.12%, respectively. The recovery was 96.95% with RSD values less than 3.24% (Supplementary Materials, Table S1).

## 3. Discussion

Bufalin (BFL), the dominant bioactive component in toad venom, has been demonstrated to possess a variety of potential pharmacological effects. However, the poor water-solubility and bioavailability limits its therapeutic application. In our previous investigations on delivery system of BFL, a novel bufalin-peptide-dendrimer inclusion (BPDI) was synthesized to enlarge the bioavailability of BFL. In the present paper, the intestinal permeability and transport capability of BPDI was evaluated using a human Caco-2 cell monolayer model. Caco-2 cells were derived from human colon adenocarcinoma. They can spontaneously differentiate into epithelium-like cells and form brush-like edges with the defined boundary and tight junctions under the culture condition. Their morphological features, functional expression of marker enzymes and osmotic characteristics are similar to those of normal intestinal epithelial cells. Moreover, the typical hydrolase and transporters for nutrients in the intestine microvillus are also expressed in Caco-2 cells. Therefore, Caco-2 cells are widely used as cell models for the in vitro study of drug transportation and metabolism of epidermal cells in small intestine [21,22].

In our current study, the Caco-2 cell monolayer was successfully established and validated by measuring the TEER values between AP and BL sides and *P*_app_ values of propranolol and atenolol, respectively. Subsequently, the permeability of BFL (*P*_app_ values) in free form and in BPDI form was detected by the developed method.

Drug molecules penetrate across Caco-2 monolayers mainly in passive diffusion, carrier-mediated influx, and efflux manners [23,24]. Based on Figure 2, transports of BFL in either free or BPDI form increased approximately in time- and concentration-dependent manner, which indicated that passive diffusion is the main transport mechanism of free BFL and BPDI. In addition, *P*_app_ values of free BFL and BPDI (Table 2 and Table 3) showed no increment with the concentration and time, as well as the ratios (*P*_app A→B_/*P*_app B→A_) were less than 1.5, which also indicated BFL and its PD inclusion transported Caco-2 monolayer mainly by passive diffusion [25,26].

As shown in Table 2 and Table 3, the *P*_app_ values of BFL increased in BPDI form, which indicated the intestinal permeability of BFL could be improved while BFL was encapsulated in BPDI form. This permeability improvement might be associated with the promotion of dissolution of BFL in BPDI form. Being a lipophilic compound, BFL passes across cytomembrane mainly in a passive transport manner, and therefore dissolution is the key factor to affect its absorption. The PD could promote the dissolution of BFL outside the cell membrane and permeability across the cell membrane.

In summary, BPDI could increase the intestinal permeability of BFL across Caco-2 monolayer, therefore PD may be a potential delivery system of water-insoluble drugs such as BFL.

## 4. Materials and Methods

### 4.1. Chemicals and Reagents

Bufalin (purity > 98%) was purchased from Chengdu PufeiDeBiotech Co., Ltd. (Chengdu, China). BPDI (content of bufalin was of 18.5%, *w*/*w*) was synthesized by Jing Jing, one co-author of current manuscript. Dimethyl sulfoxide (DMSO), propranolol and atenolol (purities > 98%), hydroxyethylpiperazine ethane sulfonic acid (HEPES), nonessential amino acid (NEAA) and ethylenediaminetetraacetic acid (EDTA) were products of Sigma-Aldrich Chemicals (Steinheim, Germany). Dulbecco’s modified Eagle’s medium (DMEM), Hanks’ Balanced Salts solution (HBSS), Eagle’s Balanced Salts solution (EBSS), fetal bovine serum (FBS) and penicillin (100 U/mL)/streptomycin were purchased from Gibco Laboratories (Life Science Technologies, Carlsbad, CA, USA). Trypsin was purchased from Shenzhen New Top Biotech Co., Ltd. (Shenzhen, China). Phosphate buffered saline (PBS) was purchased from BOSTER biological Technology Co., Ltd. (Wuhan, China). 1-octyl sodium sulfonate was product from Beijing J&K Chemical Technology Co., Ltd. (Beijing, China). Analytical grade sodium bicarbonate (NaHCO_3_) and potassium dihydrogen phosphate (KH_2_PO_4_) were purchased from Tianjin Mao Tai Chemical Reagent Factory (Tianjin, China). Liquid chromatography-grade acetonitrile, methanol, and phosphoric acid (H_3_PO_4_) were purchased from E. Merck (Darmstadt, Germany). Double deionized water was purified by Milli-Q water system (Millipore Corp., Bedford, MA, USA).

Caco-2 cells (HTB-37™) were purchased from the American Type Culture Collection (ATCC, Rockville, MD, USA), and cultured in DMEM containing d-glucose (4.5 g/L), Na_2_CO_3_ (3.7 g/L), supplemented with 10% FBS, 1% NEAA, penicillin (100 U/mL), and streptomycin (100 μg/mL) in an atmosphere of 5% CO_2_ and 90% relative humidity at 37 °C [27]. All cells were between passages 35 and 45.

### 4.2. Cytotoxicity of BPDI in Caco-2 Cells by MTT Assay

The cytotoxicities of free BFL and its formulation, BPDI against Caco-2 cells were tested using the 3-(4,5-dimethylthiazol-2-yl)-2,5-diphenyltetrazolium bromide (MTT) assay. Caco-2 cells at the logarithmic growth were seeded in 96-well plate at the concentration of 4.0 × 10^5^ per well and incubated in an atmosphere of 5% CO_2_ and 90% relative humidity at 37 °C for three days. After cells attachment, HBSS solution (negative control), paclitaxel (positive control, 25, 50, 75, and 100 μM), BFL (50, 75, 100, and 125 μM), and BPDI (containing BFL 50, 75, 100, and 125 μM) solutions were added into each well, respectively. After cells were treated for three hours, HBSS solution was removed, and cells were rinsed with PBS and incubated with 20 μL of MTT solution (0.5 mg/mL) for 4 h at 37°C and 5% CO_2_. Then the cells were shaken with 200 μL of DMSO for 10 min, and the absorbance was read on the microplate reader (SPECTROstar Omega, BMG LabTech GmbH, Ortenberg, Germany) at 570 nm. The viability rates were calculated by the equation: % = A/A_0_ × 100, wherein A was the average absorbance value of treatment group, and A_0_ was the average absorbance value of negative control group.

### 4.3. Transport Experiment of BFL and BPDI through Caco-2 Monolayer

#### 4.3.1. Establishment of Caco-2 Monolayer Model

Caco-2 cells (4.0 × 10^5^ per mL) at the logarithmic growth were seeded into the AP (apical) side of a 24-well Transwell™ plates (Corning Inc., Cambridge, MA, USA). DMEM solution was added into the apical (AP) side (0.5 mL) and basal (BL) side (1.0 mL) of the chamber, respectively. The cell culture was maintained at 37 °C under 90% humidity and 5% CO_2_. The medium was refreshed every two days in the first week, and then on every day the following week. After cells were cultured for 21 days, the integrity of the cell monolayer was evaluated by measuring trans-epithelial electrical resistance (TEER) values between AP and BL sides with Millicell-ERS system and by transport experiment using the standard compounds, propranolol and atenolol (the high and poor transcellular transport markers), respectively [28,29]. The cell inserts were considered as qualified for trans-membrane transport evaluation only if the resistance reached above 500 Ω/cm.

#### 4.3.2. Transport Experiment

On day 21, the culture medium was removed from the AP and BL sides of the trans-well filters. The Caco-2 monolayers were rinsed twice with pre-warmed HBSS (37 °C) and incubated for 20 min. Then HBSS was removed completely. The test solution of BFL or BPDI (was added to AP (0.4 mL) or BL side (3.2 mL), and then same volume of HBSS was added to the corresponding sides (BL or AP sides). Incubation continued at 37 °C for 180 min. To evaluate the permeability of test compounds across the cell monolayer, solutions from BL (0.4 mL) or AP (3.2 mL) side were collected at different time intervals (60, 120, and 180 min), and replaced with the same volume of HBSS. The collected solutions were lyophilized and stored under −80 °C before HPLC analysis. Experiments for each time point were repeated three times and were performed on the independent chamber.

### 4.4. HPLC Analysis

#### 4.4.1. Chromatographic Conditions

Chromatographic analysis was performed using a Alltima TM C_18_ column (4.6× 250 mm, 5 μm) on an Agilent 1200 liquid chromatography system, equipped with a quaternary solvent delivery system, an auto-sampler and a DAD detector. The isocratic eluting solution consists of A: 0.2% phosphoric acid and B: acetonitrile (54:46, *v*/*v*); the detection wavelength and flow rate were set at 226 nm and 1.0 mL/min, respectively. The HPLC analysis will be run for 25 min.

#### 4.4.2. Preparation of BFL Standard Solution and Test Solutions

Accurately weighed 3.86 mg of BFL was dissolved in 10 mL HBSS to create a stock standard solution with concentration of 1000 μM and then stored at −80 °C away from light. Then the stock solution was diluted with fresh HBSS to a series of concentrations ranged of 0.2–20.0 μM to establish the calibration curve. All samples were dissolved in 150 μL methanol and centrifuged at 13,000 rpm for 5 min, respectively. The supernatants were collected for HPLC analysis.

#### 4.4.3. Validation of HPLC Method

The HPLC method was validated in terms of linearity and precision and accuracy. Three concentrations of BFL standard in six replicates in a single day were analyzed as intra-day precision and duplicating the intra-day experiment on two successive days was analyzed as inter-day precision. The recoveries were calculated by comparing the analytical amounts of BFL at three concentrations with real amounts of added BFL standards.

### 4.5. Data Analysis

Apparent permeability values (*P*_app_) for the test compound was calculated according to the equation [22]

*P*_app_ = (*dQ*/*dt*)/(*AC*_0_)

where *P*_app_ is the apparent permeability (cm/s), *dQ*/*dt* is the appearance rate of the test compound on the receiver side (μg/s), *A* represents the surface area of carbon ester membrane (cm^2^), and C_0_ is the initial concentration of compounds on the given sides (μg/L). The results presented in this study are the averages of at least three replicates and were expressed as the mean ± standard deviation (SD).

## Figures and Tables

**Table 1:** Cytotoxicities of BFL and BPDI in Caco-2 cells.

Compounds	Viability (%)			
Paclitaxel	25 μM	50 μM	75 μM	100 μM
	65.3 ± 7.2	43.5 ± 5.4	34.8 ± 3.2	30.5 ± 2.6
BFL	50 μM	75 μM	100 μM	125 μM
	102.06 ± 7.2	100.10 ± 8.6	99.09 ± 8.5	98.90 ± 6.4
BPDI	50 μM	75 μM	100 μM	125 μM
	103.28 ± 10.2	98.60 ± 8.2	100.75 ± 4.8	97.20 ± 9.8

**Table 2:** The apparent permeability coefficient (*P*_app_) values of bi-directional transport of BFL and BPDI with different concentrations.

Concentration (μM)	*P*_app_ (10^−6^cm/s)				*P*_app AP→BL_/*P*_app BL→AP_	
	AP→BL		BL→AP			
	BFL	BPDI	BFL	BPDI	BFL	BPDI
50	1.58 ± 0.32	5.48 ± 0.42	1.37 ± 0.18	3.83 ± 0.70	1.15	1.43
75	1.47 ± 0.08	4.65 ± 0.48	1.32 ± 0.14	3.39 ± 0.53	1.11	1.37
100	1.46 ± 0.02	4.22 ± 0.39	1.29 ± 0.10	3.32 ± 0.12	1.13	1.27
125	1.64 ± 0.12	4.34 ± 0.48	1.35 ± 0.45	3.21 ± 0.82	1.21	1.35

AP→BL: Transport from AP to BL side; BL→AP: from BL to AP; *P*_app AP→BL_/*P*_app BL→AP_: the ratio of *P*_app BL→AP_ to *P*_app AP→BL_. The time point was at 120 min. Data are means ± S.D. (*n* = 3).

**Table 3:** *P*_app_ values of bi-directional transport of BFL and BPDI with time course.

Time (min)	*P*_app_ (10^−6^cm/s)				*P*_app AP→BL_/*P*_app BL→AP_	
	AP→BL		BL→AP			
	BFL	BPDI	BFL	BPDI	BFL	BPDI
60	1.52 ± 0.22	5.24 ± 0.82	1.14 ± 0.28	3.91 ± 0.82	1.33	1.40
120	1.36 ± 0.08	4.40 ± 0.84	1.08 ± 0.17	3.20 ± 0.74	1.26	1.38
180	1.28 ± 0.02	4.18 ± 0.64	0.99 ± 0.12	2.97 ± 0.17	1.30	1.40

AP→BL: Transport from AP to BL side; BL→AP: from BL to AP; *P*_app AP→BL_/*P*_app BL→AP_: the ratio of *P*_app BL→AP_ to *P*_app AP→BL_. The concentrations of BFL and BPDI were 75 μM, respectively. Data are means ± S.D. (*n* = 3).

## Supplementary Materials

Supplementary materials are available online. Table S1: The intra-and inter-day precision and recovery of BFL.
