# Supplementary material for: Enhanced Intestinal Permeability of Bufalin by a Novel Bufalin-Peptide-Dendrimer Inclusion through Caco-2 Cell Monolayer
Source: Molecules. 2017 Nov 29;22(12):2088. doi: 10.3390/molecules22122088 (PMC6149814; doi:10.3390/molecules22122088)
Supplement: Supplementary file 1 [file molecules-22-02088-s001.pdf]

Table S. The intra-and inter-day precision and recovery of BFL

Standard samples (μM)	Intra-day		Inter-day		Mean recovery [b] (%)
	Mean (μM)	RSD [a] (%)	Mean (μM)	RSD (%)	
1	0.98	4.2	0.96	3.9	104.32
5	4.85	3.4	4.75	2.7	96.25
10	9.65	2.1	9.58	1.8	97.54

[a] RSD (%) = (SD of amount detected/mean of amount detected) × 100

[b] Recovery (%) = 100 × (detected amount – original amount)/spiked amount, data was presented as mean of three experiments
